# Study of the reverse transition in pipe flow

**DOI:** 10.1038/s41598-023-39585-6

**Published:** 2023-07-30

**Authors:** Hikaru Yokoo, Mizuki Yamamoto, Takumi Matsumoto, Takahiro Yamada, Takeshi Kanda

**Affiliations:** 1 Chubu University, Kasugai, Aichi 487-8501 Japan; 2 Present Address: Mie University, Mie, Japan; 3 Present Address: KVK Co., Ltd., Gifu, Japan

**Keywords:** Fluid dynamics, Mechanical engineering, Engineering, Physics

## Abstract

In the reverse transition in pipe flow, turbulent flow changes to less disturbed laminar flow. The entropy of the flow appears to decrease. This study examined the reverse transition experimentally and theoretically using entropy change and momentum balance models, not in terms of disturbance in the flow. The reverse transition was accomplished by decreasing the Reynolds number. The transitions approximately correlated with local Reynolds numbers. The initial Reynolds number of the transition became larger, and the pressure at low Reynolds numbers was greater than in ordinary pipe flow. These behaviours were caused by turbulent flow in the pipe undergoing a reverse transition. We showed that the entropy did not decrease in the reverse transition by including the entropy due to friction in the development region.

## Introduction

The laminar-to-turbulent transition was first described by Reynolds in the nineteenth century^1^, and since that time it has been studied in pipe and duct flows. Although the transition phenomenon is common and apparently simple, several problems remain to be solved. One of the problems is the occurrence of “relaminarization”, also known as a reverse transition^2–9^. In this phenomenon, disturbed turbulent flow changes to less disturbed laminar flow. Consequently, the entropy of the flow appears to decrease. Narasimha and Sreenivasan^2^ reported that “a common reaction when the subject was mentioned used to be that the implied transition from disorder to order was thermodynamically impossible!” Patel and Head^3^ examined the similarities and differences in reverse transitions in pipe flows and boundary layers. Sibulkin^6^ reported that the relaminarizing transition occurred more rapidly at smaller Reynolds numbers. Narayanan^7^ reported the distance required for the reverse transition. Seki and Matsubara^8^ discussed the critical Reynolds number in the case of relaminarization. These studies realized a reverse transition by decreasing the Reynolds number to less than the critical Reynolds number, which was reported to range from 1400 to 1700. Below the critical Reynolds number, there is no transition from laminar to turbulent flow. The reverse transition has been discussed in terms of the dissipation of disturbance. However, there has been no answer to the question whether the reverse transition appears to violate the second law of thermodynamics.

Kanda^10^ studied a typical laminar-to-turbulent transition in straight-pipe flow by momentum balance in the transition region. Hattori et al.^11^ revealed that the inflow turbulence from the development region into the transition region affected the downstream transition condition by entropy change, not in terms of disturbance. These relationships are fundamental in physics even when flow is laminar or turbulent, regardless of whether there is disturbance.

In the present study, the reverse transition in pipe flow was examined experimentally and theoretically. The condition for a reverse transition and the laminar-to-turbulent transition in pipe flow undergoing a reverse transition were examined using entropy change and momentum balance models. This paper shows the experimental and analytical results.

## Experimental setup

**Figure 1:**
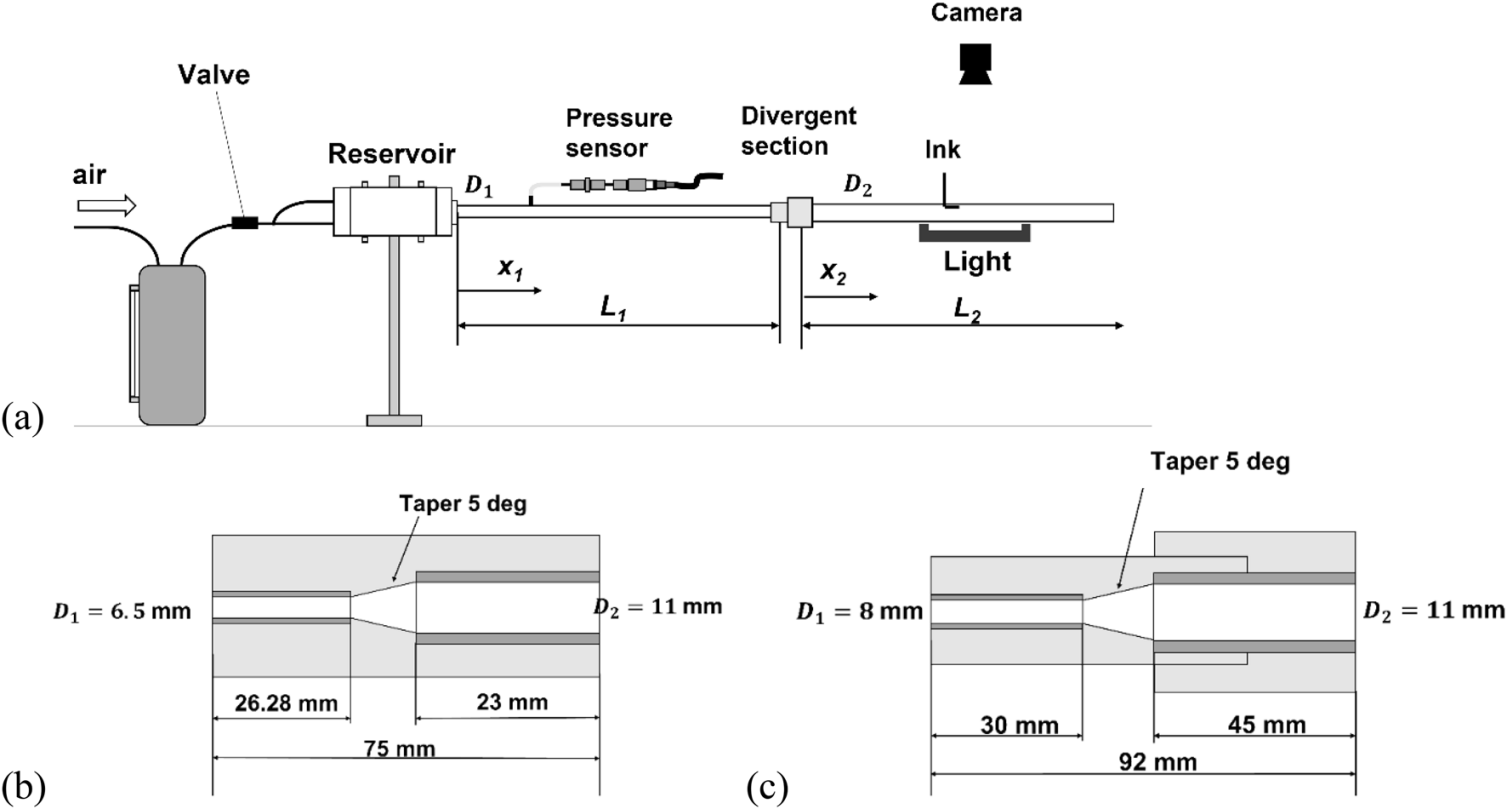
Experimental setup. (**a**) Schematic of the experimental setup, (**b**) divergent pipe block of Pipe A, and (**c**) divergent pipe block of Pipe B.

The pipe flow conditions were monitored through ink visualization and pressure measurement. Two urethane pipes with different diameters were connected by a divergent duct. Figure 1 shows schematics of the experimental setup. Two urethane pipes with different diameters were connected by a divergent duct.

In this situation, the Reynolds number was smaller in the downstream pipe than in the upstream pipe. To investigate the influence of the divergent ratio of the upstream/downstream pipes on the reverse transition, two sets of connected pipes were tested. In Pipe A, a pipe with an inner diameter of *D*_1_ = 6.5 mm and a length of *L*_1_ = 1.73 m was connected to a downstream pipe with an inner diameter of *D*_2_ = 11 mm and a length of *L*_2_ = 2.13 m. In Pipe B, a pipe with an inner diameter of *D*_1_ = 8 mm and a length of *L*_1_ = 2.13 m was used for the upstream pipe. For comparison, the downstream pipe was tested alone as an ordinary pipe, designated Pipe C.

The urethane tubes were not quite straight. The effect of the wavy pipe was verified in preliminary tests using a curved passage with an inner diameter of 6.5 mm and a length of 4.9 m. Though a 33-mm radius of curvature affected the pipe flow conditions, a 330-mm did not affect the flow conditions; the pressures and visualized flow conditions were the same as those of the straight pipe. Therefore, the present pipe configuration is considered sufficient to observe the typical transition conditions.

Room temperature water was supplied from a reservoir. The Reynolds number, *Re*_*D*_, was calculated by measuring the water mass over 5 or 10 s with an AND EK-3000i weight scale (Yamato Scientific Co. Ltd, Japan), which has the measurement error of ± 0.1%. For visual observation, ink was injected at several positions along the pipe inner wall surface using a fine stainless-steel tube with an outer diameter of 0.5 mm. Photographs were taken with the FLIR Blackfly S USB3.0 camera at an exposure time of 6 μs. The pressure was measured using PGM-02 KG and PGM-1kG sensors and the EDX-10B/14A measurement system (all from Kyowa Co., Ltd., Japan). The measurement error is ± 0.5%.

## Results

**Figure 2:**
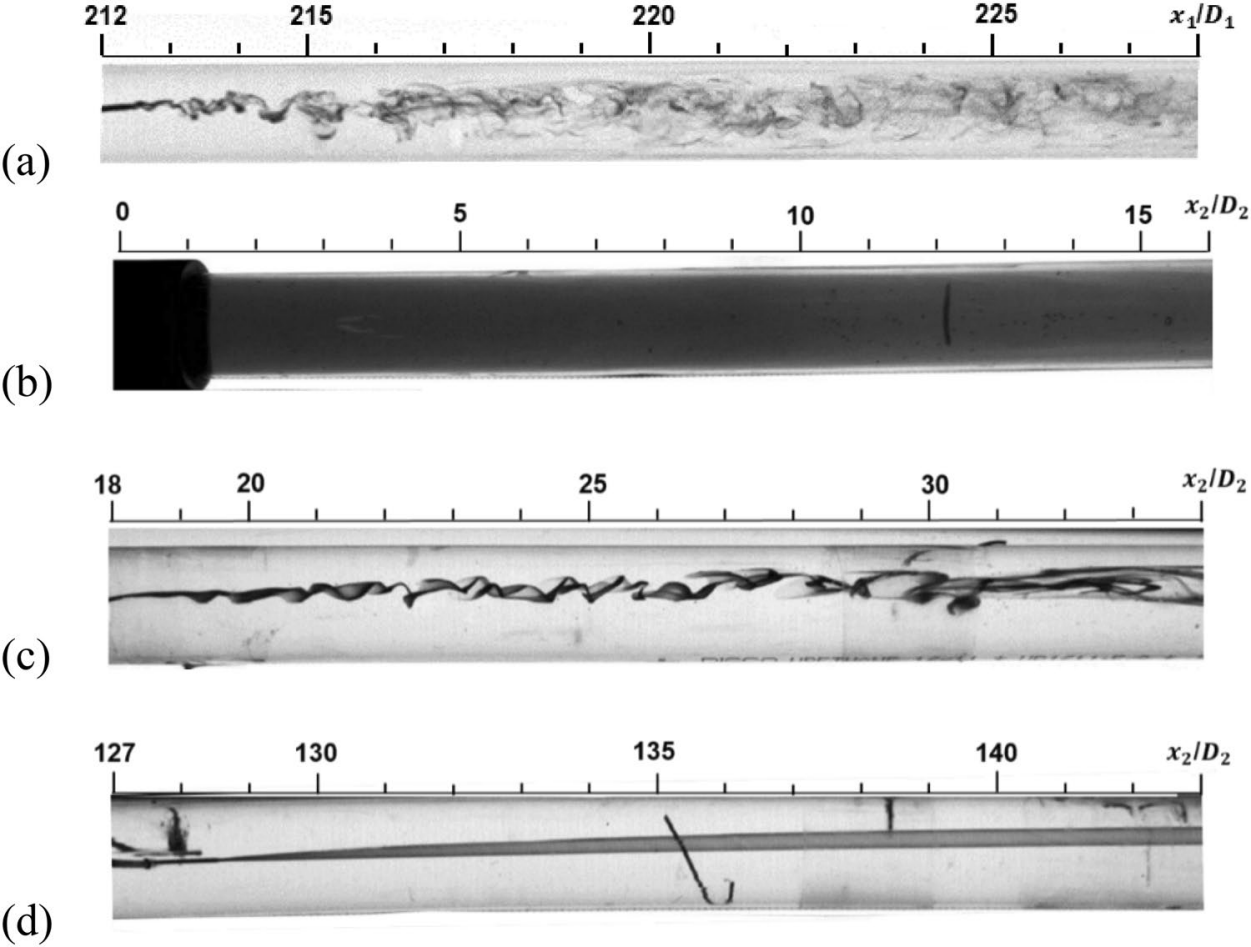
Flow visualization at *Re*_*D*_ = 3540 in the upstream pipe and *Re*_*D*_ = 2090 in the downstream pipe of Pipe A: (**a**) *x*_1_/*D*_1_ = 212.3; (**b**) ink injection at *x*_1_/*D*_1_ = 212.3 and photograph at the divergent section exit; (**c**) *x*_2_/*D*_2_ = 18.2; (**d**) *x*_2_/*D*_2_ = 127.3.

Figure 2 shows the flow conditions upstream and downstream of the divergent duct of Pipe A. The Reynolds number was *Re*_*D*_ = 3540 in the upstream pipe and *Re*_*D*_ = 2090 in the downstream pipe. The ink flows moved from left to right. The subscript 1 indicated the upstream pipe, and 2 indicated the downstream pipe. Each *x*-coordinate was the distance from the entrance of each upstream/downstream pipe. The rod visible in the figure was stainless-steel wire that plugged the hole used for pressure measurement, which did not affect the flow.

**Figure 3:**
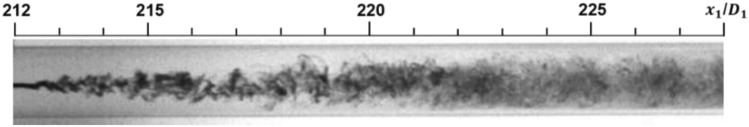
Flow visualization at *Re*_*D*_ = 7320 in the upstream pipe at *x*_1_/*D*_1_ = 212.3 of Pipe A.

In Fig. 2a, a constant vortex appeared, and the flow underwent a transition. Figure 2b shows the flow condition at the exit of the divergent section. The black area on the left-hand side was the divergent section. Ink injected from *x*_1_/*D*_1_ = 212.3 in the upstream pipe thoroughly diffused. The disturbed flow condition resembled the results of previous pipe flow studies, including divergent sections or a sudden expansion^12–14^. The disturbed flow condition also resembled turbulent flow at Reynolds numbers larger than 7000 (Fig. 3). According to the previous study^11^, the pipe flow becomes turbulent under the condition; ink diffuses quickly and each vortex cannot be identified anymore.

In Fig. 2, downstream of the divergent section, the large-scale flow structure became clear at *x*_2_/*D*_2_ = 18.2. There was no waviness in Fig. 2d at *x*_2_/*D*_2_ = 127.3. The flow far downstream of the divergent section was laminar. The reverse transition occurred at *Re*_*D*_ = 2090. This was larger than previously reported values of critical Reynolds numbers^8,15,16^.

**Figure 4:**
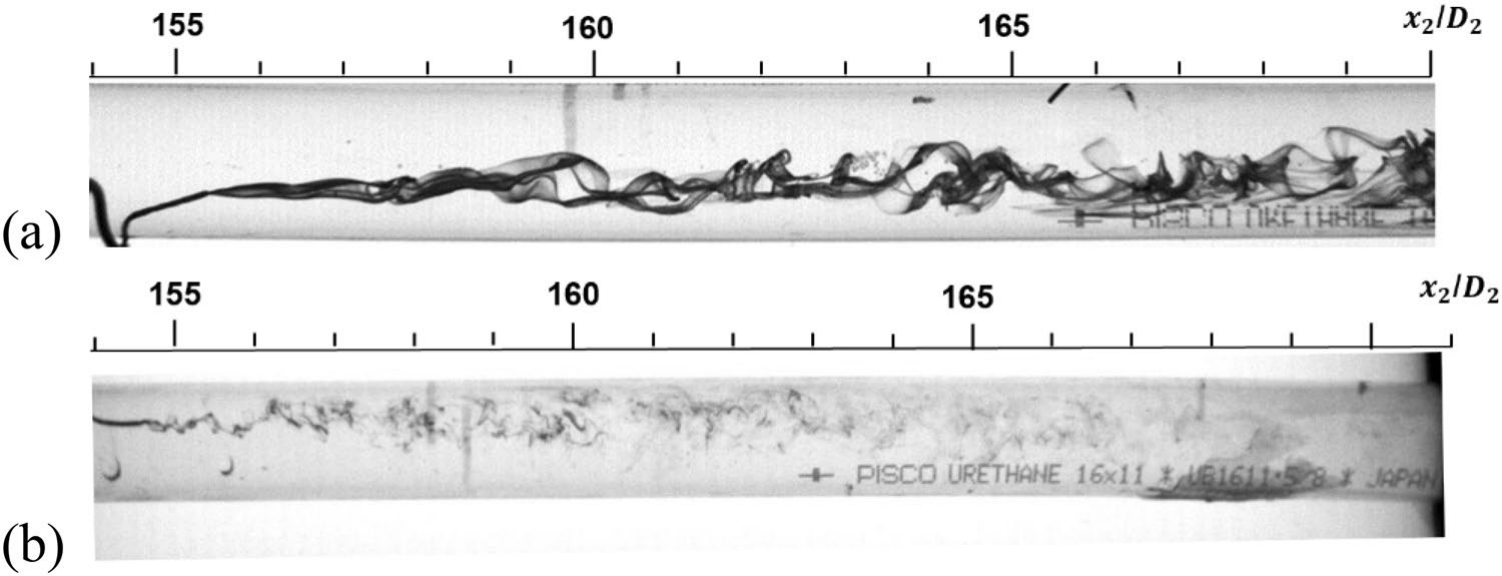
Flow visualization in the downstream pipe of Pipe A. *x*_2_/*D*_2_ = 154. (**a**) *Re*_*D*_ = 2740, (**b**) *Re*_*D*_ = 4330.

Figure 4a shows large-scale flow structure far downstream of the divergent section at *Re*_*D*_ = 2740 in the downstream pipe of Pipe A. This structure appeared at *Re*_*D*_ = 2000–4000. Above *Re*_*D*_ = 4000, numerous and small vortices appeared (Fig. 4b). Under these conditions, ink also thoroughly diffused at the exit of the divergent section.

In the experiments, the disordered flow became more ordered after a certain distance downstream. The reverse transition was accomplished by decreasing the Reynolds number. The transitions were approximately correlated with the local Reynolds numbers. The reverse transitions did not depend on the ratio of pipe diameter to length. Various studies have examined divergent pipe flow or sudden expansion pipe flow^12–14,17–20^. If those studies had examined flow condition farther downstream, the reverse transition would have been observed. In the downstream pipe, however, the laminar flow continued until *Re*_*D*_ = 2000, and the beginning of the transition was delayed. Wavy or slowly fluctuating flow appeared after the laminar flow condition at *Re*_*D*_ = 2000–3000, which appeared at *Re*_*D*_ = 1200–2300 in the ordinary pipe flow transition^11^. Slug flow behaviour did not appear, which appeared in the ordinary pipe flow.

**Figure 5:**
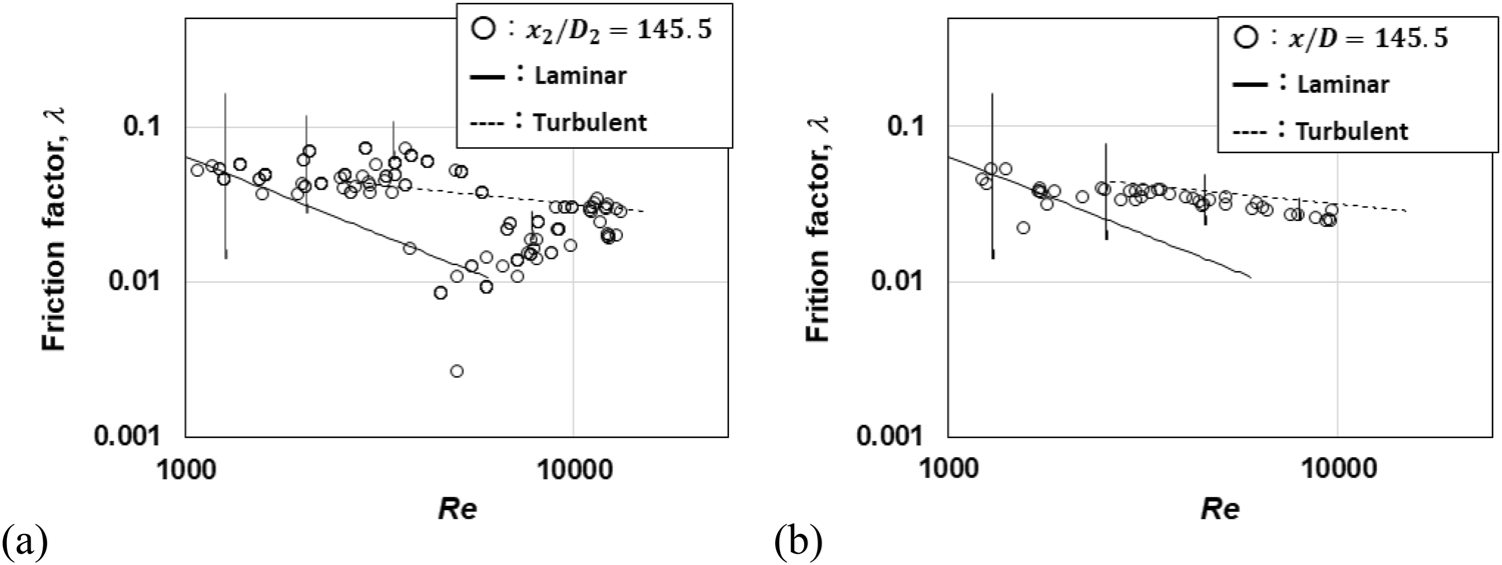
Friction factor calculated with measured pressure: (**a**) Pipe A; (**b**) Pipe C.

Figure 5 presents the measured pressure of the downstream pipe of Pipe A and that of Pipe C in the form of the friction factor, $\lambda$. The pressure was measured at *x*_2_/*D*_2_ = 145.5. The pipe end was open to the atmosphere. For comparison, the Darcy friction factor of the laminar pipe flow (Laminar) and the Blasius formula of the turbulent pipe flow (Turbulent)^21^ are also plotted.

In Pipe C, the factor decreased at approximately *Re*_*D*_ = 1600. In a previous study, the factor deceased between *Re*_*D*_ = 1200 and 12,000^11^. Patel and Head reported that the coefficient deviated at *Re*_*D*_ = 1700 and returned at *Re*_*D*_ = 30,000^22^. In the downstream pipe, however, the friction factor decreased between *Re*_*D*_ = 4000 and 10,000. The decrease started at a higher Reynolds number than expected. However, the factor was approximately 0.01 greater than the theoretical value for *Re*_*D*_ = 2000–4000. They were not observed in the ordinary pipe configuration.

**Figure 6:**
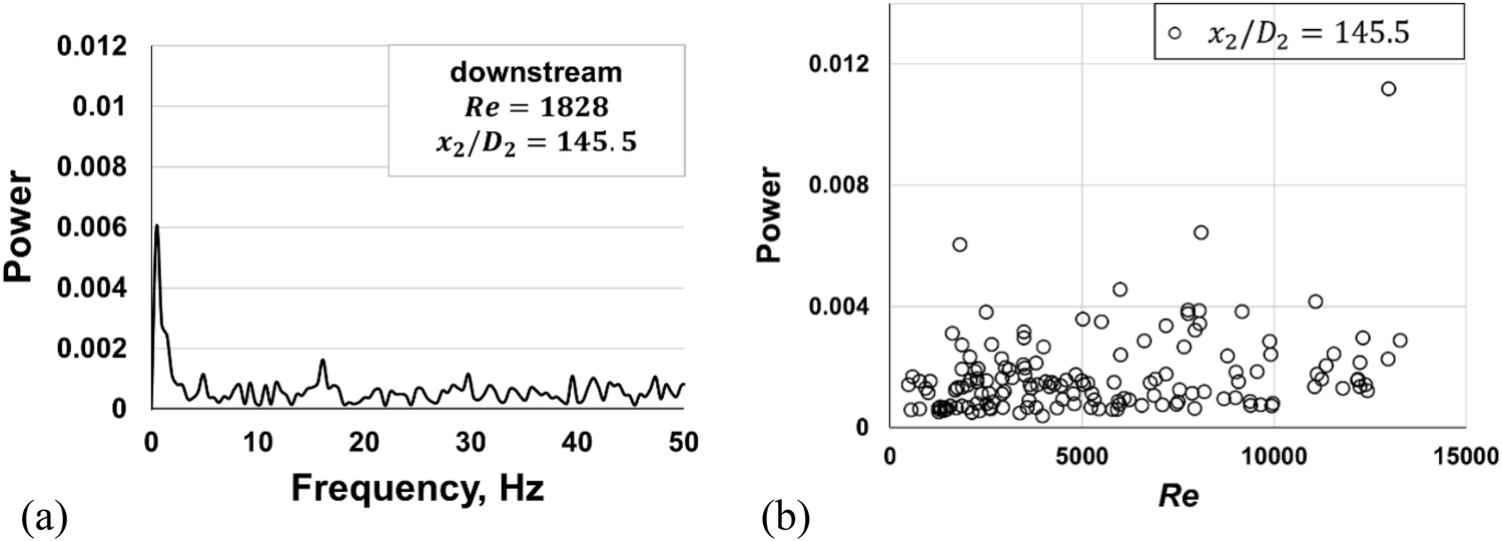
FFT plot of pressure measured at *x*_2_/*D*_2_ = 145.5 of the downstream pipe of Pipe A. (**a**) FFT plot; (**b**) distribution of maximum FFT power.

Figures 6 shows fast Fourier transform (FFT) plot at the downstream pipe of Pipe A. Although most FFT diagrams show no peaks at any frequency, the power at some given frequencies increased as shown in Fig. 6a. Figure 6b shows the distribution of the maximum power against the Reynolds number of the downstream pipe. Power peaks exist at Reynolds numbers of approximately 2000, 8000, and 13,000. The appearance of a power peak at a particular Reynolds number indicates that a large change in the flow structure occurs at this value^11^. The Reynolds numbers at the peak power values in Pipe B were almost the same as those in Pipe A. This similarity indicates that the flow conditions do not greatly depend on the ratio of pipe diameter to length. In Pipe C and the ordinary pipe^11^, the peak appeared at *Re*_*D*_ = 1200. The flow condition for the transition was different from that in ordinary pipe flow at low Reynolds numbers.

## Discussion

### Entropy change model and ordinary transition

The flow was very disturbed at the divergent exit; in other words, there was no special distribution in velocity. This condition resembled fluid in a reservoir. The flow from the divergent section was presumed to be a mixture of the average-velocity-profile flow from the reservoir and turbulent flow. The ordinary pipe flow transition was discussed in a previous study using an entropy change estimated on a pipe cross section^11^. In the present study, first, mass-weighted entropy change under the ordinary transition is discussed.

The velocity profile of a laminar pipe flow is

1 $${u}_{l}={u}_{max,l}\left\{1-{\left(\frac{r}{R}\right)}^{2}\right\},$$

where *R* is the radius of the pipe, *r* is the distance from the centre of the pipe, and *u*_*max*_ is the maximum velocity at the centre of the pipe. The kinetic energy of the laminar pipe flow, *E*_*k,l*_, is

2 $${E}_{k,l}=\rho \cdot 2\pi {\int }_{0}^{R}\frac{1}{2}{\left[{u}_{max,l}\left\{1-{\left(\frac{r}{R}\right)}^{2}\right\}\right]}^{3}r\cdot dr=\rho \overline{u}\frac{\pi {D }^{2}}{4}\cdot 2\cdot \frac{1}{2}{\overline{u} }^{2}.$$

The average velocity is defined as $\overline{u }=\dot{m}/\left(\rho \cdot A\right)$. $\dot{m}$ is a mass flow rate, $\rho$ is density and *A* is a pipe cross section. The maximum velocity of the laminar pipe flow is ${u}_{max,l}=2\overline{u }$. For turbulent flow, the velocity profile is expressed as^23^

3 $$\frac{{u}_{t}}{{u}_{max,t}}={\left(\frac{{r}^{\prime}}{R}\right)}^{1/n},$$

where *r*' is the distance from the pipe circumference to the centre. The subscript *l* indicates the laminar flow condition, and *t* indicates the turbulent flow condition. When *n* = 7, the kinetic energy of the turbulent flow, *E*_*k,t*_, is

4 $${E}_{k,t}=\rho \cdot 2\pi {\int }_{0}^{R}\frac{1}{2}{\left\{{u}_{max,t}{\left(\frac{{r}^{\prime}}{R}\right)}^{1/7}\right\}}^{3}\left(R-{r}^{\prime}\right)d{r}^{\prime}=\rho \overline{u}\frac{\pi {D }^{2}}{4}\cdot 1.057\cdot \frac{1}{2}{\overline{u} }^{2}.$$

The maximum velocity of the turbulent flow is ${u}_{max,t}=1.224\overline{u }$.

The static thermal energy is derived from the total energy by subtracting the kinetic energy. The static thermal energy of the laminar flow is expressed in the form of static temperature.

5 $${T}_{l}={T}_{0}-\frac{1}{2c}\cdot 2{\overline{u} }^{2}={T}_{0}-\frac{1}{2c}\cdot 2{\left(\frac{{Re}_{D}\cdot \mu }{\rho D}\right)}^{2}.$$

Here, *c* is a specific heat, *T*_0_ is total temperature and *μ* is viscosity. The subscript 0 indicates the total condition. The static temperature in the laminar or turbulent flow is different from that in the average-velocity-profile flow. Therefore, the entropy change in the laminar or turbulent flow is different from that in average-velocity-profile flow. The entropy change from the average-velocity-profile flow to the laminar flow, *Δs*_1-*l*_, is expressed as:

6 $$\frac{\Delta {s}_{ave-l}}{c}=\mathit{ln}\left(\frac{{T}_{l}}{{T}_{ave}}\right)=\mathit{ln}\left\{\frac{{T}_{0}}{{T}_{ave}}-\frac{2}{2}\frac{{{Re}_{D}}^{2}}{{{Re}_{{T}_{ave}}}^{2}}\right\}\approx \mathit{ln}\left(1-\frac{{{Re}_{D}}^{2}}{{{Re}_{T1}}^{2}}\right)=\frac{\Delta {s}_{1-l}}{c}<0.$$

The subscript 1 indicates the condition at the entrance to the downstream pipe. Here, ${Re}_{T1}=\rho D\sqrt{c{T}_{1}}/\mu$. At *T*_0_ = 290 K, *c* = 4183 J/kg K, *μ* = 0.001 Pa s, and *D* = 10 mm; *Re*_*T*0_ = 1.10 × 10^7^ for water. The entropy change from the average-velocity-profile flow to the turbulent flow, $\Delta {s}_{1-t}/c$, is expressed as:

7 $$\frac{\Delta {s}_{1-t}}{c}=\mathit{ln}\left(\frac{{T}_{t}}{{T}_{1}}\right)\approx \mathit{ln}\left\{1-\frac{1.057}{2}\cdot \frac{{{Re}_{D}}^{2}}{{{Re}_{T1}}^{2}}\right\}.$$

In the development region from the reservoir to the establishment of the pipe flow velocity profile, the entropy change is caused by friction. It is calculated using an average of a formula for the drag coefficient of the boundary layer, *C*_*D*_, and a pipe flow friction factor, $\lambda$^21^. The friction work under the boundary layer is as follows:

8 $${w}_{frc}=\frac{{F}_{frc}\cdot \overline{u}}{\dot{m}}\approx \frac{\pi DL\cdot {c }_{D}\frac{1}{2}\rho {\overline{u} }^{2}\cdot \overline{u}}{\frac{\pi }{4}{D }^{2}\rho \overline{u} }=\frac{L}{D}\cdot 4{c}_{D}\cdot \frac{1}{2}{\overline{u} }^{2}.$$

The change of entropy in the development region, *Δs*_*frc,l*_, is:

9 $$\begin{aligned} \frac{{\Delta s_{{frc,l}} }}{c} & = {\text{ln}}\left( {1 + \frac{{w_{{frc,l}} }}{c}} \right) \approx {\text{ln}}\left( {1 + \frac{{L_{{dv,l}} }}{D} \cdot \frac{{4c_{D} + \lambda _{l} }}{2} \cdot \frac{1}{2}\frac{{Re_{D} ^{2} }}{{Re_{{T1}} ^{2} }}} \right) \\ & = {\text{ln}}\left\{ {1 + \frac{{L_{{dv,l}} }}{D} \cdot \frac{1}{4}\left( {4 \cdot \frac{{1.328}}{{\sqrt {Re_{D} } }}\sqrt {\frac{D}{{L_{{dv,l}} }}} + \frac{{64}}{{Re_{D} }}} \right) \cdot \frac{{Re_{D} ^{2} }}{{Re_{{T1}} ^{2} }}} \right\} > 0, \\ \end{aligned}$$

where *L*_*dv*_ is the length of the development region.

The flow condition under the minimum increase in entropy, i.e., 0, is examined. The entropy change from the average velocity profile to the laminar flow condition in the ordinary transition is expressed as:

10 $$\left(\Delta {s}_{1-l}/c\right)+\left(\Delta {s}_{frc,l}/c\right)=0.$$

Substituting Eqs. (6) and (9) into Eq. (10) and using a Taylor expansion, the following equation is derived:

11 $$\frac{{{Re}_{D}}^{2}}{{{Re}_{T1}}^{2}}\left\{-1+\frac{{L}_{dv,l}}{D}\cdot \left(\frac{1.328}{\sqrt{{Re}_{D}}}\sqrt{\frac{D}{{L}_{dv,l}}}+\frac{16}{{Re}_{D}}\right)\right\}\approx 0.$$

From Eq. (11), the relation between the development length of the laminar flow, ${L}_{dv,l}$, and the Reynolds number is derived as follows:

12 $$\frac{{L}_{dv,l}}{D}\approx 0.0449\cdot {Re}_{D}.$$

Here, the boundary layer Reynolds number has a relation to the pipe flow Reynolds number:

13 $${Re}_{x}\approx {Re}_{D}\frac{x}{D}.$$

When *Re*_*x*_ in the development region becomes the transition Reynolds number ${Re}_{x,tr}$ of 3 × 10^5^, turbulence begins to flow into the transition region. The Reynolds number of the pipe flow is calculated using Eqs. (11) and (13) as follows:

14 $${Re}_{D}\approx \sqrt{\frac{{Re}_{x,tr}}{0.0449}}=4.7187\sqrt{{Re}_{x,tr}}=3.985\sqrt{3\times {10}^{5}}=2580.$$

This number approximately agrees with the ordinary laminar-to-turbulent flow transition Reynolds number *Re*_*D*_ = 2400 of Pipe C.

When the flow in the development region is turbulent,

15 $$\frac{\Delta {s}_{1-t}}{c}+\frac{\Delta {s}_{frc,t}}{c}=0.$$

The entropy increase caused by turbulent friction is as follows;

16 $$\begin{aligned} \frac{{\Delta s_{{frc,t}} }}{c} = & ln\left( {1 + \frac{{w_{{frc,t}} }}{c}} \right) \approx {\text{ln}}\left( {1 + \frac{{L_{{dv,t}} }}{D} \cdot \frac{{4c_{D} + \lambda _{t} }}{2} \cdot \frac{1}{2}\frac{{Re_{D} ^{2} }}{{Re_{{T1}} ^{2} }}} \right) \\ = & {\text{ln}}\left( {1 + \frac{{L_{{dv,t}} }}{D} \cdot \frac{1}{2}\left\{ {4\frac{{0.0303}}{{Re_{{x,tr,e}} ^{{1/7}} }} + \frac{{0.3164}}{{Re_{D} ^{{1/4}} }}} \right\} \cdot \frac{1}{2}\frac{{Re_{D} ^{2} }}{{Re_{{T1}} ^{2} }}} \right) \\ \end{aligned}$$

Substituting Eqs. (7) and (16) into Eq. (15), the entropy change from the average-velocity-profile to the turbulent flow condition is expressed as:

17 $$\frac{{{Re}_{D}}^{2}}{{{Re}_{T1}}^{2}}\left\{-\frac{1.057}{2}+\frac{{L}_{dv,t}}{D}\cdot \frac{1}{4}\left\{4\frac{0.0303}{{{Re}_{x,tr,e}}^{1/7}}+\frac{0.3164}{{{Re}_{D}}^{1/4}}\right\}\right\}\approx 0$$

The Reynolds number at the end of the boundary layer transition, *Re*_*x,tr,e*_, is approximately twice the Reynolds number at the starting point of the transition of *Re*_*x,tr,i*_ = 3 × 10^5^, i.e., *Re*_*x,tr,e*_ ≈ 6 × 10^5^^24^. Using Eq. (13) and using ${Re}_{x,tr,e}$ of 6 × 10^5^, the transition Reynolds number is calculated to be 13,480. It agrees with finally-transitioned turbulent Reynolds number of approximately 12,000^11^ and the Reynolds number at the FFT power peak.

Rewriting Eq. (17) using Eq. (13), we obtain the development length in the turbulent flow, ${L}_{dv,t}$, as follows.

18 $$\frac{0.3164}{{{Re}_{D}}^{1/4}}\cdot \frac{{L}_{dv,t}}{D}+4\frac{0.0303}{{{Re}_{D}}^{1/7}}{\left(\frac{{L}_{dv,t}}{D}\right)}^{6/7}-2\times 1.057\approx 0$$

When the boundary layer transition ends in the development region at *Re*_*x,tr,e*_ ≈ 6 × 10^5^, the turbulent flow begins to flow into the transition region. Using the drag coefficients of the laminar and the turbulent boundary layers and the same entropy change procedure, the transition Reynolds number is calculated to be 6700, which agrees with the Reynolds numbers at the flow structure change and the FFT peak.

### Reverse-transition and its delay at small Reynolds numbers

Change from average velocity profile flow to reverse transitioned laminar flow of the mixed flow is an ordinary development in laminar pipe flow. The other change is examined; change from turbulent to reverse-transitioned laminar flow. When the turbulent flow enters the downstream pipe and becomes laminar, the entropy change is expressed as:

19 $$\left(\Delta {s}_{1-l}/c\right)+\left(\Delta {s}_{frc,t}/c\right)=\left(\Delta {s}_{1-t}/c\right).$$

In Eq. (19), the final condition is laminar flow, $\Delta {s}_{1-l}/c$, whereas the inflow is turbulent.

The entropy change in the mixed flow from the condition at the exit of the divergent section to the reverse-transitioned laminar flow is:

20 $$\frac{\Delta {s}_{1-l}}{c}=\left(1-\alpha \right)\left\{-\frac{\Delta {s}_{frc,l}}{c}+0\right\}+\alpha \left\{-\frac{\Delta {s}_{frc,t}}{c}+\frac{\Delta {s}_{1-t}}{c}\right\},$$

where *α* is the ratio of turbulent flow. The left-hand side represents the entropy change at the final reverse transition condition. The right-hand side is the entropy change of the mixed flow. Even if local entropy decreases, at the same time, if the entropy of a whole system does not decrease, then the second law of thermodynamics is not violated. By including the entropy change in a whole flow system, the second law of thermodynamics is not violated, even in the reverse transition. Equation (20) can be rewritten using Eqs. (6) , (7), (9), and (16) as

21 $$\left(1-\mathrm{\alpha }\right)\left(\frac{1.328}{\sqrt{{Re}_{x,tr}}}+\frac{16}{{Re}_{D}}\right)+\mathrm{\alpha }\left(\left\{\frac{0.0303}{{{Re}_{x,tr}}^{1/7}}+\frac{0.3164}{4{{Re}_{D}}^{1/4}}\right\}+\frac{{Re}_{D}}{{Re}_{x,tr}}\frac{1.057}{2}\right)\approx \frac{{Re}_{D}}{{Re}_{x,tr}}$$

The transition Reynolds number, *Re*_*D*_, is calculated using Eq. (21) with respect to *α*. According to Eq. (21), the turbulence causes the transition Reynolds number of the pipe flow to increase in the downstream pipe as *α* increases. The large-scale flow structure (Fig. 5a) appeared at approximately *Re*_*D*_ = 3000 in the downstream pipe of Pipes A and B. In the ordinary transition, this structure appeared at approximately *Re*_*D,tr*_ = 2400. When the structure appeared, the turbulence produced in the development region flowed into the transition region^11^. The beginning of the transition was delayed in the reverse transition. The turbulent flow ratio is estimated to be *α* = 0.2.

**Figure 7:**
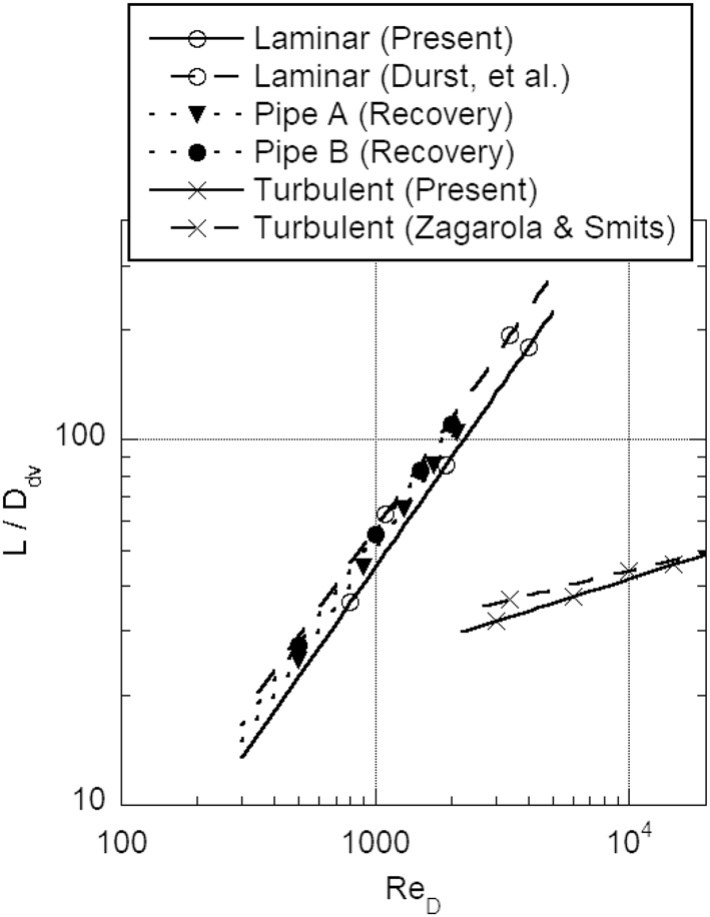
Development length ratios of laminar and turbulent pipe flows and laminar recovery length ratio of Pipes A and B.

The ratios of the development length to diameter of the laminar and turbulent flows calculated with Eqs. (12) and (18) are compared with the empirical results by Durst et al. (Laminar)^25^ and Zagarola and Smits (Turbulent)^26^ in Fig. 7. Each calculated ratio agrees well with laminar or turbulent empirical result. In the figure, the ratios of laminar recovery length to diameter measured in Pipes A and B are also plotted. The flows became laminar up to a downstream Reynolds number of approximately 2100. Under this flow condition, the turbulence flowing into the downstream pipe will be small, with *α* ≈ 0. Therefore, the ratio of the recovery length to the diameter was almost the same as the ratio of the development length in the ordinary laminar pipe flow.

At large Reynolds numbers, turbulent flow enters the transition region from the development region even in the ordinary transition, and the final pipe flow condition is also turbulent. Therefore, the reverse transition condition is the same as that of the ordinary transition flow at large Reynolds numbers.

### Lower/higher friction factors in transition

In ordinary pipe flow, the dynamic pressure becomes static pressure in the laminar-to-turbulent transition because the momentum of the laminar flow is greater than that of the turbulent flow^10,11^. This change starts approximately at *Re*_*D*_ = 1200 in the ordinary transition. The momentum of the laminar pipe flow, *F*_*l*_, is

22 $${F}_{l}=\rho \cdot 2\pi {\int }_{0}^{R}{\left[{u}_{max,l}\left\{1-{\left(\frac{r}{R}\right)}^{2}\right\}\right]}^{2}r\cdot dr=1.333\rho {\overline{u} }^{2}\pi {\left(\frac{D}{2}\right)}^{2}.$$

The momentum of the turbulent flow, *F*_*t*_, is

23 $${F}_{t}=\rho \cdot 2\pi {\int }_{0}^{R}{\left\{{u}_{max,t}{\left(\frac{{r}^{\prime}}{R}\right)}^{1/7}\right\}}^{2}\left(R-{r}^{\prime}\right)d{r}^{\prime}=1.020\rho {\overline{u} }^{2}\pi {\left(\frac{D}{2}\right)}^{2}.$$

When the change from momentum to pressure occurred in region *L*_*l*–t_, the momentum change of the laminar and turbulent flows, $\Delta {F}_{l-t}$, was equal to the pressure change, expressed by the difference of friction factors between laminar and turbulent flows:

24 $$\frac{D}{{L}_{l-t}}\cdot \frac{2}{\rho {\overline{u} }^{2}}\cdot \frac{\Delta {F}_{l-t}}{\left(\pi {D}^{2}/4\right)}=\frac{D}{{L}_{l-t}}\cdot \frac{2}{\rho {\overline{u} }^{2}}\Delta p=\Delta \overline{\uplambda }={\overline{\lambda }}_{t}-{\overline{\lambda }}_{l}=\frac{0.3164}{{{Re}_{D}}^{1/4}}-\frac{64}{{Re}_{D}}.$$

This change was estimated to be approximately 0.02 in the form of the friction factor for *Re*_*D*_ = 2000–3000 with Eq. (24). This locally increased pressure made fluid flow out of the reservoir with a smaller supply pressure^11^. The decrease in the friction factor was caused by this dynamic-to-static pressure change. The FFT peak power appeared at *Re*_*D*_ = 2000, whereas 1200 at Pipe C and the ordinary transition. This peak corresponds to the momentum change and the transition delayed in the reverse-transition.

The flow from the divergent section was a mixture of turbulent flow and average-velocity-profile flow. This mixed flow underwent a reverse transition. When the turbulent component became laminar, the static pressure was converted to the dynamic pressure of the laminar flow. A larger static pressure was required to supply the dynamic pressure to the laminar flow. In the reverse transition, the locally large friction factor in the range of *Re*_*D*_ = 2000–4000 was thus caused by this turbulent-to-laminar transition. Under these conditions, the laminar-to-turbulent transition did not appear or appeared to a small degree. Therefore, the transition was delayed in the downstream pipe.

## Conclusions

The reverse transition and subsequent transitions were studied experimentally and theoretically using entropy change and momentum balance models. We showed that the reverse transition was accomplished by decreasing the Reynolds number. The transitions approximately correlated with local Reynolds numbers. At low Reynolds numbers, the laminar-to-turbulent transition was delayed, and pressure increased in the downstream pipe. These behaviours were caused by inflow turbulence. We answered the traditional fluid dynamics question: the entropy of the flow appeared to decrease in the reverse transition. By including friction, we showed that the reverse transition did not violate the second law of thermodynamics. This study not only answered this traditional question in fluid dynamics but also showed another way to utilize fluid dynamics.

## Data Availability

If a reader needs data used in this study, the authors are ready to supply the data under a formal request with suitable reasons. Please have a contact with T. Kanda (kanda-t@isc.chubu.ac.jp).
